# Bidirectional communication between spinal cord injury and gut microbiota, from the bench to the bedside

**DOI:** 10.3389/fimmu.2026.1742885

**Published:** 2026-06-19

**Authors:** Zhaoyang Yin, Ge Gong, Jian Yin

**Affiliations:** 1 Department of Orthopedics, The Affiliated Lianyungang Hospital of Xuzhou Medical University (The First People’s Hospital of Lianyungang), Lianyungang, China; 2 Department of Geriatrics, Jinling Hospital, Affiliated Hospital of Medical School, Nanjing University, Nanjing, China; 3 Department of Orthopedics, The Affiliated Jiangning Hospital of Nanjing Medical University, Nanjing, China

**Keywords:** biomarkers, gut microbiota, inflammation, nerve, spinal cord injury

## Abstract

Spinal cord injury (SCI) is a type of central nervous system damage that often results in motor, sensory, and autonomic dysfunction, and can lead to death, with currently no effective treatment available. As research on the microbiome in central nervous system disorders progresses, the role of gut microbiota in spinal cord injury has garnered significant attention. Spinal cord injury disrupts intestinal function and triggers an imbalance in gut microbiota, while metabolites produced by gut microbiota can cross the blood-spinal cord barrier into the central nervous system, exacerbating neuroinflammation in the spinal cord. The relationship between gut microbiota dysbiosis and spinal cord injury is bidirectional. In recent years, the proposal of the ‘gut microbiota-gut-spinal cord’ axis theory has led to increased interest in the impact of gut microbiota on spinal cord injury. Gut microbiota not only serve as biomarkers for the severity of spinal cord injury but also represent potential therapeutic targets. Current research primarily focuses on the alterations in gut microbiota following spinal cord injury and the potential effects of microbiota-derived metabolites—such as aryl hydrocarbon receptor agonists and short-chain fatty acids—on secondary inflammatory responses post-injury. Although numerous studies have utilized various approaches to modulate gut microbiota to promote functional recovery after spinal cord injury, standardized and effective clinical treatments remain elusive. This review synthesizes laboratory and clinical perspectives on the mechanisms underlying the interaction between spinal cord injury and gut microbiota, aiming to provide novel insights for the therapy of spinal cord injury.

## 1 Introduction

Spinal cord injury (SCI) is a devastating form of central nervous system trauma that frequently results in permanent paralysis, sexual dysfunction, and bladder/bowel dysfunction (1, 2). Globally, the incidence rate varies from 15 to 40 cases per million in high-income countries, with approximately 133,000 to 226,000 new cases reported each year worldwide (3). SCI severely compromises the quality of life for patients and significantly increases healthcare expenditures. Most spinal cord injuries are caused by high-energy trauma, such as traffic accidents, falls from height, or competitive sports, while some cases may arise from non-traumatic factors, including tumors or infections. The pathophysiology of SCI consists of two main components. The initial mechanical damage, referred to as primary injury, occurs immediately after the injury and involves direct cell death, disruption of vascular structures, and hemorrhage due to mechanical trauma. This primary injury subsequently triggers complex secondary damage, primarily characterized by intense neuroinflammatory responses, oxidative stress, and the formation of glial scars (4, 5). Although early interventions, such as spinal cord decompression, corticosteroid administration, and hyperbaric oxygen therapy, have become common clinical practices, the complexity of the underlying pathophysiological mechanisms poses a significant challenge to SCI treatment (6).

Patients with SCI experience numerous post-injury complications, with digestive system dysfunction resulting from the disruption of the regulatory axis between the gut and central nervous system being particularly prominent. This includes conditions such as autonomic dysreflexia, intestinal motility disorders, constipation, and fecal incontinence (7–9). Furthermore, the high-dose antibiotics administered to SCI patients severely disrupt the balance of gut microbiota. Recent evidence has increasingly demonstrated a bidirectional relationship between the central nervous system and gut microbiota (10). Damage to the central nervous system impairs intestinal peristalsis and barrier function, consequently affecting microbial survival, bacterial translocation, and the immune responses of gut immune cells (11). Conversely, disruption of the gut microbiota can trigger the secretion of pro-inflammatory factors and neurotoxic substances, further contributing to neurological disorders that extend beyond spinal cord-brain injuries, including Parkinson’s disease, multiple sclerosis (12–14), and autism (15). Studies have confirmed a significant reduction in butyrate-producing bacteria following SCI, accompanied by a marked increase in the secretion of pro-inflammatory cytokines such as TNF-α, IL-1β, IL-6, and IFN-γ (16, 17). The bidirectional regulatory relationship between the gut microbiota and the spinal nervous system is referred to as the spinal cord-microbiota-gut axis. The following sections will elaborate on the regulatory mechanisms between these two systems from both laboratory and clinical research perspectives.

This review systematically summarizes the latest evidence from preclinical laboratory investigations and emerging clinical observations, and establishes a translational chain ranging from mechanistic exploration to clinical intervention. This study comprehensively sorts out multiple targeted microbiota modulation strategies, including probiotics, organic acids, cytokines, microecological intervention, polyphenols, fatty acids, FMT, EAW, as well as Bifidobacterium. Furthermore, it provides critical and objective insights into the current translational bottlenecks, safety risks, research limitations and unresolved challenges existing in this research field.

## 2 The impact of spinal cord injury on gut microbiota in laboratory studies

The gut microbiota encompasses the diverse community of bacteria, viruses, and eukaryotic microorganisms residing in the colon, stomach, and small intestine, commonly referred to as gut flora. Maintaining a dynamic equilibrium of gut microbiota is essential for the body’s development and metabolic processes; conversely, an imbalance can precipitate a range of diseases (18). Research indicates that in healthy adults, there is a higher relative abundance of *Ruminococcus* and *Bacteroides* (19). Additionally, bacteria from the genera *Blautia* and *Faecalibacterium* are recognized for their capacity to mitigate inflammation (20, 21). The sympathetic preganglionic neurons that govern the small intestine and colon are predominantly located within the T5–T10 segments of the thoracic spinal cord. When spinal cord injury occurs above the T5 level, it results in the disruption of brain control over the spinal autonomic neural networks that regulate intestinal function (22, 23). SCI leads to the disruption of normal sympathetic nerve control in the intestines, resulting in impaired intestinal motility, secretion, and immune function. These alterations subsequently cause changes in the gut microbiota (24). The study revealed that spinal cord injury adversely affected the metabolism of glutamine, D-glutamate, and taurine, as well as aminoacyl-tRNA biosynthesis within the intestinal tract of mice (25).

Alpha (α)-diversity indices evaluate intrasample gut microbiota diversity, covering species richness and evenness. The Shannon index integrates species quantity and distribution uniformity, with higher values representing greater microbial diversity. The Simpson index reflects dominant species concentration; values near 1 denote balanced species distribution and high diversity, while those close to 0 indicate dominant species prevalence and low diversity. *Chao1* index estimates actual species number and is sensitive to rare taxa, enabling accurate quantification of low-abundance species to better characterize species richness. Similar to *Chao1*, *ACE index* also predicts total species richness, yet it weights abundant species more heavily while taking rare species into account (26, 27). Beta (β) diversity evaluates inter-sample variations in gut microbiota composition, revealing microbial community similarities and disparities across different individuals or time points. Typical analytical approaches include PCoA, a dimensionality reduction method that maps microbiota data into 2D or 3D scatter plots. Shorter inter-sample distances denote higher compositional similarity, and this method enables intuitive observation of sample clustering characteristics. As another mainstream dimensionality reduction technique, NMDS prioritizes maintaining relative sample distances via iterative optimization to match original high-dimensional spatial relationships, facilitating visual comparison of microbial community discrepancies. Differently, PCA conducts linear transformation to extract core principal components retaining major original data information, which can screen key bacterial taxa driving inter-group microbiota differences and visualize sample distribution via score plots (28, 29).

Some scholars suggest that SCI can lead to alterations in microbiome β-diversity but does not affect the α-diversity of gut microbiota. In a mouse hemisection SCI model, α-diversity index analysis revealed no significant changes in the Shannon index or bacterial community richness between the pre- and post-SCI groups. However, PERMANOVA analysis of β-diversity and community composition demonstrated significant differences in fecal microbial communities between the two groups (30). At the phylum level, the composition of fecal microbiota prior to spinal cord injury was ranked as follows: *Bacteroidetes* (65.1%), Firmicutes (33.9%), *Proteobacteria* (0.42%), *Actinobacteria* (0.24%), and *Tenericutes* (0.15%). Post-spinal cord injury, the ranking changed to: *Firmicutes* (50%), *Bacteroidetes* (48.3%), *Proteobacteria* (0.66%), *Tenericutes* (0.37%), and *Actinobacteria* (0.33%). Interestingly, the *Firmicutes*/*Bacteroidetes* ratio showed no significant difference between pre- and post-injury mice. Taxonomic analysis of bacterial families revealed that in pre-spinal cord injury mouse fecal samples, the relative abundance of bacterial families was ranked as follows: *Muribaculaceae* (53.1%), *Lachnospiraceae* (14.58%), *Lactobacillaceae* (10.76%), *Ruminococcaceae* (7.4%), and *Rikenellaceae* (7.24%). Post-spinal cord injury, the abundance of *Bacteroidales S24–7* was relatively low (35.73%), while *Lachnospiraceae* (23.62%) and *Lactobacillaceae* (18.84%) showed higher proportions. Based on the LDA effect size algorithm (LEfSe) analysis, *Prevotellaceae UCG-001*, *Ruminococcaceae UCG-013*, and *Marvinbryantia* exhibited higher LDA scores prior to spinal cord injury. Conversely, *Lactobacillus*, *Lachnospiraceae NK4A136* group, *Alistipes*, and *Blautia* showed enrichment after SCI (30).

However, another study suggested that SCI appears to reduce the α-diversity of microbial communities. A mouse model of traumatic spinal cord injury at the T8-T10 segments demonstrated a significantly decreased Chao1 index and observed species index in the SCI group. The Shannon index and Simpson index also exhibited declining trends, accompanied by a reduced Pielou’s evenness index in the SCI group (31). These discrepancies may arise from variations in animal models, detection time points, and analytical methods. β-diversity analysis similarly indicated that spinal cord injury led to gut microbiota dysbiosis. Post-SCI, the abundance of *Firmicutes* and *Proteobacteria* increased, while the abundance of *Bacteroidetes* decreased. The *Firmicutes/Bacteroidetes* (F/B) ratio serves as an indicator of gut microbiota dysbiosis, and the SCI group exhibited a significantly elevated F/B ratio. At the genus level, the SCI group showed lower relative abundances of *Lactobacillus*, *Allobaculum*, and *Succinivibrio*, but higher relative abundances of *Shigella* and *Bacteroides*. Overall, pro-inflammatory bacteria (e.g., *Shigella*, *Bacteroides*, *Rikenella*, *Staphylococcus*, and *Mucispirillum*) significantly increased following SCI, while anti-inflammatory bacteria (e.g., *Lactobacillus*, *Allobaculum*, and *Sutterella*) markedly decreased. Furthermore, gut bacterial-derived metabolites also exhibited significant differences after spinal cord injury. The excessive accumulation of five metabolites—L-leucine, L-methionine, L-phenylalanine, L-isoleucine, and L-valine—post-SCI may induce oxidative stress and inflammatory responses, thereby contributing to secondary injury following spinal cord injury.

## 3 The impact of spinal cord injury on gut microbiota in clinical research

Patients with SCI exhibit a distinct gut microbiota composition, characterized by a reduced abundance of butyrate-producing bacteria (32), such as Faecalibacterium, and an increased abundance of inflammation-associated bacteria (33). In contrast to laboratory studies, clinical research environments are considerably more complex; however, clinical data more accurately reflect real-world human conditions. An Israeli clinical cohort study enrolled 63 participants, comprising 31 SCI patients and 32 non-SCI controls. No significant differences were observed in age, sex, or body mass index between the two groups. The SCI patients were further categorized into subacute (n=13) and chronic (n=18) subgroups. All participants were not subjected to any dietary restrictions. All patients with SCI experienced symptoms of constipation and relied on various treatments, including stimulant laxatives, mild laxatives, and enemas, to facilitate bowel movements. Antibiotics are frequently administered to SCI patients, with the subacute SCI group demonstrating significantly higher usage rates of formula supplements compared to the chronic SCI group, alongside decreased albumin levels. An analysis of intestinal microbial populations revealed a significant reduction in Firmicutes content among chronic SCI patients when compared to both the control group and the final samples from the subacute group. The subacute SCI group exhibited an increased content of *Verrucomicrobia* relative to the control group. The spinal cord injury group displayed an increasing trend in 54 amplicon sequence variants (ASVs), including *Faecalibacterium prausnitzii*, *Prevotella copri*, and *Ruminococcus gnavus*. Overall, the degree of gut microbial species variation in the subacute group was intermediate between that of the control group and chronic patients. Notably, the American Spinal Injuries Association (ASIA) score, C-reactive protein (CRP), hemoglobin (HGB), and albumin all demonstrated significant associations with microbial variations to varying extents (34).

Compared to non-athletes, athletes exhibit increased energy expenditure across various substrates, resulting in distinct metabolic characteristics (35). A study conducted at the Swiss Paraplegic Centre in Nottwil, Switzerland, included 11 elite athletes with SCI who had participated in international competitions such as para-cycling, wheelchair athletics, and wheelchair tennis. Analysis of gut microbiota revealed differences in bacterial taxa associated with the duration and frequency of weekly training, as well as waist circumference. Longer training durations positively correlated with *Akkermansia* and *Akkermansiaceae*, while negatively correlating with *Prevotellaceae*. Additionally, *Muribaculaceae* was negatively correlated with the average number of weekly training sessions, and waist circumference demonstrated a negative correlation with *Butyricimonas*. Significant differences in α-diversity were observed concerning gender, gastrointestinal quality of life index (GIQLI) scores, total caloric intake, total fat intake, total carbohydrate intake, and high-sensitivity C-reactive protein (hs-CRP). Variations in β-diversity were associated with impaired gut sympathetic nervous system function and correlated with HbA1c at the phylum level. The 16S rRNA sequencing of gut microbiota in SCI athletes revealed that the most prevalent and abundant bacterial phylum was Firmicutes, with the most common genera being *Blautia*, *Ruminococcus*, *Faecalibacterium*, and *Bacteroides*. Among SCI athletes, the most prevalent and abundant families included *Ruminococcaceae*, *Lachnospiraceae*, *Bacteroidaceae*, *Oscillospiraceae*, *Peptostreptococcaceae*, and *Christensenellaceae*. However, significant differences exist in the gut microbiota among various subgroups of SCI athletes (36).

## 4 The impact of gut microbiota modulation on spinal cord injury in laboratory studies

The biomolecular cascade of secondary injury following SCI significantly impacts neurological function (37). Peripheral immune factors migrate to the spinal cord, releasing neurotoxic substances that lead to persistent neuroinflammation after SCI (38). The inflammatory response post-SCI plays a dual role in the recovery process; early high-dose hormone therapy may induce immunodeficiency syndrome, increasing the incidence of infection and mortality (39, 40). Altered permeability of the blood-spinal cord barrier and intestinal mucosal barrier after SCI allows metabolites from the gut microbiota to cross into the central nervous system, triggering neuroinflammation (2, 41, 42). These metabolites include short-chain fatty acids (SCFAs), peptidoglycan branches, chain amino acids, and neurotransmitters such as serotonin, acetylcholine, dopamine, and γ-aminobutyric acid (43). Additionally, *Clostridium*, a type of bacteria that proliferates under abnormal conditions, often hinders recovery from SCI. Conversely, an increase in beneficial gut bacteria, such as *Bifidobacterium*, which produces SCFAs, contributes to the restoration of neurological function following SCI (11).

Qiuyu Cen et al. (44) found that following SCI, the abundance of *Firmicutes* decreased while that of *Bacteroidetes* increased in rats. Additionally, the abundance of *Lactobacillaceae* significantly decreased, whereas harmful families such as *Peptostreptococcaceae* were significantly enriched. At the genus level, the abundances of opportunistic pathogens such as *Clostridioides*, *Faecalibaculum*, *Blautia*, and *Staphylococcus* significantly increased, while the abundance of the probiotic genus *Lactobacillus* markedly decreased. Notably, the abundance of *Limosilactobacillus reuteri* decreased substantially after injury. Administration of *Limosilactobacillus reuteri (L. reuteri)* DSM 17938 intervention can restore gut microbiota homeostasis, generate indole-3-aldehyde (IAld) through tryptophan metabolism, and simultaneously reduce the level of the neurotoxic metabolite quinolinic acid. IAld, acting as a ligand, activates the aryl hydrocarbon receptor (AhR) signaling pathway in the spinal cord and intestine, which upregulates downstream CYP1A1 expression, thereby enhancing the expression of intestinal tight junction proteins (ZO-1, Occludin, Claudin-1). This process repairs the damaged intestinal barrier and reduces the spread of intestinal inflammation. Furthermore, this bacterium significantly inhibits the polarization of microglia toward the pro-inflammatory M1 phenotype, reduces the levels of key pro-inflammatory cytokines such as IL-1β, IL-6, and TNF-α, alleviates local neuroinflammation in the spinal cord, and promotes recovery after SCI.

Indole-3-propionic acid (IPA) is another organic acid and a tryptophan-derived metabolite produced by the gut microbiota, known for its potent anti-inflammatory effects in neurological disorders (45). Recent studies have demonstrated that gut microbiota-derived IPA reduces inflammatory factors such as IL-1β, IL-6, CCL2, iNOS, and COX-2 by activating the aryl hydrocarbon receptor (AhR) and inhibiting the NF-κB and MAPK signaling pathways. This mechanism suppresses excessive astrocyte activation and neuroinflammation, thereby alleviating secondary spinal cord injury in mice and promoting recovery of motor function (46).

Ursolic acid (UA), a plant-derived pentacyclic triterpene, exhibits anti-inflammatory, antioxidant, and neuroprotective activities (47, 48). Studies have shown that UA increases the relative abundance of *Muribaculaceae*, *Lachnospiraceae_NK4A136_*group, and *Alloprevotella genera* in the gut of SCI mice. Additionally, it reverses disruptions in intestinal glutamine and D-glutamine metabolism, nitrogen metabolism, aminoacyl-tRNA biosynthesis, as well as taurine and hypotaurine metabolism, thereby alleviating inflammatory responses and promoting synaptic regeneration in SCI mice (25). Researchers suggest that inducing an anti-inflammatory macrophage phenotype contributes to recovery from SCI.

IL-13 not only regulates macrophage polarization but also exerts neuroprotective effects (49). Studies indicate that systemic application of recombinant IL-13 (rIL-13) significantly enhances the recovery of neurological function following spinal cord injury. Analysis of gut microbiota revealed that rIL-13 treatment increased the abundance of Clostridiales vadin BB60 and *Acetitomaculum*, while decreasing the relative abundance of Anaeroplasma, Ruminiclostridium_6, *Ruminococcaceae UCG-013*, and *Ruminococcaceae UCG-010*. Notably, regardless of treatment group, *Clostridiales vadin BB60* exhibited a significant positive correlation with functional recovery in mice after spinal cord injury (30). Overall, rIL-13 therapy induced an anti-inflammatory shift in gut microbiota, contributing to the alleviation of SCI.

Some researchers have utilized fecal microbiota transplantation (FMT) from healthy mice as a therapeutic approach for spinal cord injury. They discovered that FMT enhances intestinal barrier integrity and mitigates gut inflammation by downregulating the IL-1β/NF-κB signaling pathway, thereby facilitating axonal regeneration in mice (50). Furthermore, FMT increases the abundance of the *Christensenellaceae* family and the *Butyricimonas genus* within the intestinal tract of mice, which are known to produce elevated levels of SCFAs. Additionally, FMT can reverse the decline of potentially beneficial bacteria such as *Blautia*, *Anaerostipes*, and *Lachnospiraceae_NK4A136*, while also reducing the proliferation of potentially harmful *Bilophila (*50). γδ T cells serve as critical inflammatory mediators during infection, exacerbating tissue inflammation through the secretion of IL-17 and IFN-γ (51, 52). They also play a pivotal role in inflammation within the central nervous system. γδ T cells can infiltrate the spinal cord injury site shortly after the injury occurs, potentially hindering the recovery of spinal cord neurological function by releasing interferon (IFN)-γ (53). IL-17 is a crucial pro-inflammatory cytokine that recruits and activates neutrophils (54). By inhibiting the release and activity of IL-17, neuroinflammation can be alleviated, thereby promoting the recovery of neurological function (55, 56). Additionally, γδ T cells exacerbate inflammatory responses and damage in ischemic brain injury through IL-17 (57). Deshuang Xi et al. (58) found that FMT treatment partially reversed the SCI-induced decrease in the abundance of *Akkermansia*, *Bacteroidetes*, *Globicatella*, *Parasutterella*, and *Rhodospirillales*, and also partially reversed the post-SCI increase in *Allobaculum*, *Olsenella*, *Sporobacter*, and *Porphyromonadaceae*. Following spinal cord injury, γδ T cells migrate from gut-associated lymphoid tissues to the spinal cord. FMT regulates γδ T cells through dendritic cell (DC)-T regulatory (Treg) cell interactions, induces metabolic reprogramming of DCs, and suppresses IL-17+ γδ T cells by promoting Treg cell induction in mesenteric lymph nodes (mLNs) post-SCI. In summary, FMT corrects gut microbiota dysbiosis following SCI, reduces γδ T cell migration from gut lymphoid tissues to the spinal cord, thereby inhibiting the accumulation of IL-17+ γδ T cells and IL-17 production in the spinal cord, ultimately promoting neurological functional recovery.

During the process of secondary injury in SCI, microglia-mediated inflammatory responses are closely associated with injury prognosis. Modulating gut microbiota to influence microglial activation has garnered significant attention (59). Numerous studies have confirmed that resveratrol exhibits anti-inflammatory (60), anti-oxidant (61) and anti-neurodegenerative effects (62). Although resveratrol can significantly inhibit microglial activation to alleviate neuroinflammation and injury, its limited ability to cross the blood-brain barrier and the blood-spinal cord barrier restricts its application in the central nervous system (63, 64). Ning He et al. (65) found that resveratrol had no significant effect on the Shannon index of gut microbiota α-diversity in SCI mice. Beta diversity analysis revealed that resveratrol reversed the SCI-induced increase in *Clostridiales* abundance, decrease in *Erysipelotrichaceae* abundance, and reduction in the relative abundances of *Dubosiella* and *Parasutterella*. Further analysis demonstrated that resveratrol restored gut microbiota composition, increased butyrate concentration, reduced lipopolysaccharide-binding protein levels, and significantly decreased concentrations of IL-6, monocyte chemoattractant protein-1 (MCP-1), and TNF, thereby suppressing microglia-induced inflammatory responses following spinal cord injury.

SCFAs serve as a critical link between the gut microbiota and the immune system, playing a vital role in stabilizing immune responses within the central nervous system and maintaining a balance between anti-inflammatory and pro-inflammatory processes (11). Following SCI, disruptions in the gut microbiota result in a decrease in SCFA-producing bacteria, which exacerbates intestinal inflammatory responses and hinders neurological recovery (11, 33). Acetate and butyrate, as prominent SCFAs, can ameliorate spinal cord injury by inhibiting the expression of inflammatory factors in microglia (66–69). Pan Liu and colleagues administered oral exogenous SCFAs in an SCI model using SD rats, demonstrating their potential to mitigate neural damage (70). Research indicates that SCFAs stabilize the intestinal microbiota, preserve villus height and mucosal thickness, promote a shift of intestinal T cells toward an anti-inflammatory phenotype, enhance the secretion of IL-10 by regulatory T cells (Tregs), and influence the balance between spinal Tregs and IL-17+ γδ T cells, thereby suppressing inflammatory responses in the spinal cord and facilitating functional recovery. Additionally, SCFAs counteract the decreased expression of IL-10 and the increased expression of IL-17 in spinal cord tissue.

Disruption of the gut-lung axis after SCI is an independent mechanism for the high incidence of pulmonary infection (71). In the murine T9 SCI model, oral administration of *Bifidobacterium longum BL300* significantly restored intestinal *Bifidobacterium* abundance and reversed gut dysbiosis induced by SCI. This probiotic intervention repaired intestinal mucosal barrier integrity by upregulating the expression of tight junction proteins ZO-1 and occludin, preserved enteric neuronal function, and reduced systemic translocation of bacterial products such as LPS and LBP. *Bifidobacterium* supplementation decreased the similarity between gut and lung microbiota, limited gut-derived pathogenic bacteria (e.g., *Enterobacteriaceae*) from colonizing the lung, and alleviated *Escherichia coli*-triggered pulmonary inflammation, inflammatory cell infiltration, and alveolar structural damage. Mechanistically, *Bifidobacterium* shifted gut microbial metabolic function toward enhanced short-chain fatty acid production and suppressed pathogen-related virulence pathways, thereby stabilizing the gut-lung axis and exerting protective effects against SCI-associated pulmonary inflammatory injury (72).

*Akkermansia muciniphila* (AKK) is a mucin-degrading bacterium found in the intestinal mucus layer of the gut microbiota. It is regarded as a next-generation gut probiotic that promotes neural repair and functional recovery. This mucin-degrading commensal strengthens intestinal mucosal integrity by upregulating tight junction proteins and mucin production, thereby limiting systemic lipopolysaccharide leakage and restraining peripheral inflammatory overflow that drives secondary spinal cord damage (73). Concurrently, AKK suppresses neuroinflammation by inhibiting microglial and astrocytic overactivation, reducing proinflammatory cytokine release, and attenuating NF-κB and NLRP3 inflammasome signaling within the injured spinal cord microenvironment (74). Through generating short-chain fatty acids, AKK activates GPR41/43 signaling, stabilizes the blood–spinal cord barrier, and enhances brain-derived neurotrophic factor expression via histone deacetylase inhibition to support neuronal survival and axonal regeneration (75). Collectively, by integrating mucosal barrier reinforcement, immune regulation, microbial metabolite signaling, and neurotransmitter homeostasis, AKK represents a promising next-generation probiotic strategy for alleviating secondary injury and promoting neural repair in spinal cord injury.

Although promising preliminary findings have emerged from preclinical animal models, significant challenges persist in translating these observations into clinical practice and therapeutic applications. Variability in the colonization efficiency of different probiotic strains, heterogeneous host responses, and divergent intervention strategies collectively constrain the generalizability of current research outcomes. Future investigations should prioritize the optimization of delivery regimens and the design of rigorous clinical trials to ultimately facilitate successful clinical translation in the field of neural repair. Laboratory study summaries are presented in **Table 1**.

**Table 1:** Laboratory studies.

Research topics	Therapeutic methods	Animal model	Cell model	Changes in microbiota	Molecular mechanism	Ref
Probiotics	L. reuteri DSM 17938	SD rat cervical spinal cord contusion model	Not involved	Dysbiosis reversed, Lactobacillus ↑, Limosilactobacillus reuteri ↑, harmful bacteria ↓	DSM17938 activates AhR signaling via tryptophan metabolism−derived IAld, restores intestinal barrier, inhibits microglial M1 polarization, and reduces IL−1β/IL−6/TNF−α to alleviate neuroinflammation	(44) Qiuyu Cen et al., 2025
Organic acid	IPA	Spinal cord contusion model at T9-T10 segments in adult female C57 BL/6 J mice	Astrocytes derived from C57 BL/6 J stimulated with TNF-α	Not involved	IPA reduces inflammatory factors such as IL-1β/IL-6/CCL2/iNOS/COX-2 by activating AhR and inhibiting NF-κB and MAPK signaling pathways, thereby suppressing excessive astrocyte activation and neuroinflammation	(46) Dapeng Yu et al., 2026
Organic acid	UA	T10 spinal cord contusion model in C57BL/6N mice	Not involved	Lachnospiraceae_NK4A136_group↑, Alloprevotella↑, Muribaculaceae↓	Ursolic acid protects neurons and promotes synaptic regeneration by improving gut microbiota composition, regulating glutamine and glutamate metabolism, enhancing nitrogen metabolism, and inhibiting the NF-κB/IL-1β/TNF-α inflammatory axis	(25) Zijie Rong et al., 2022
Cytokine	rIL-13	T8 thoracic spinal cord hemisection injury model in female BALB/cJRj mice	Not involved	Clostridiales vadin BB60↑, Acetitomaculum↑, Anaeroplasma↓, Ruminiclostridium_6↓, Ruminococcus↓	rIL-13 modulates gut microbiota composition and activates anti-inflammatory metabolic pathways (glyoxylate cycle, palmitoleic acid synthesis), promotes macrophage polarization toward anti-inflammatory phenotype, and improves motor functional recovery after spinal cord hemisection injury in mice	(30) Ibrahim Hamad et al., 2023
Microecological intervention	FMT	T10 spinal cord contusion model in female C57BL/6N mice	Not involved	Firmicutes↑, Christensenellaceae↑, Butyricimonas↑, Blautia↑, Anaerostipes↑, Lachnospiraceae_NK4A136↑, Bilophila↓	FMT reshapes gut microbiota, raises SCFA-producing bacteria abundance and SCFAs content, suppresses NF-κB signaling, and preserves intestinal barrier and spinal cord neurons	(50) Yingli Jing et al., 2021
Microecological intervention	FMT	T9-T11 spinal cord contusion model in female SD rats	Rat DCs/CD4+ T/γδ T cells coculture for Treg induction, IL-17 inhibition and DC metabolic reprogramming	Akkermansia↑, Bacteroides↑, Globicatella↑, Parasutterella↑, Allobaculum↓, Olsenella↓, Sporobacter↓	FMT remodels gut microbiota and enriches Akkermansia, induces metabolic reprogramming of regulatory dendritic cells in mesenteric lymph nodes, facilitates Treg generation, restrains the migration of intestinal-derived γδ T cells toward the spinal cord and IL-17 secretion, and alleviates neuroinflammation	(58) Deshuang Xi et al., 2024
Polyphenols	Resveratrol	T10 spinal cord contusion model in female C57BL/6 mice	LPS-induced activation model of BV2 microglia	Lactobacillus↑, Erysipelotrichales↑, Dubosiella↑, Parasutterella↑, Clostridiales↓, Lachnospiraceae_NK4A136↓	Resveratrol remodels gut microbiota, elevates the abundance of butyrate-producing bacteria and increases butyric acid levels, reduces blood LPS infiltration, and directly inhibits spinal microglial activation and neuroinflammation	(65) Ning He et al., 2022
Fatty acids	SCFAs	T9-T10 spinal cord contusion model in female SD rats	Primary Treg & γδ T cells stimulated with SCFAs/IL-10	Not involved	SCFAs induce intestinal anti-inflammatory Treg generation and promote their migration to the spinal cord, upregulate IL-10 secretion, suppress the activation of IL-17^+^ γδ T cells and IL-17 production, and alleviate neuroinflammation	(70) Pan Liu et al., 2023
Probiotics	Bifidobacterium	T9 complete spinal cord transection model	Not involved	Pulmonary: Streptococcus↑, Staphylococcus↑, Rothia↑, Aerococcus↑;α diversity increased, β diversity significantly deviated from the normal state Intestinal: Bifidobacterium↓, Lactobacillus↓, Allobaculum↓	Bifidobacterium has the capacity to restore the gut microbiota structure, repair the intestinal mucosal barrier, reduce bacterial translocation, and exerts a lung-protective effect by stabilizing the gut-lung axis	(72) Yuanqing Ding et al., 2026

AhR, Aryl hydrocarbon receptor; FMT, Fecal microbiota transplantation; IAld, Indole-3-aldehyde; IPA, Indole-3-propionic acid; *L. reuteri, Limosilactobacillus reuteri*; rIL-13, recombinant IL-13; SCFAs, Short-chain fatty acids; SD, Sprague–Dawley; UA, Ursolic acid.

## 5 The impact of gut microbiota on spinal cord injury in clinical research

The human gut harbors a vast array of microorganisms, the majority of which are symbiotic and play a crucial role in human health. These microorganisms are influenced by various factors, including the environment, lifestyle, diet, medication, and genetics (76). A healthy gut microbiota is essential for regulating the host’s intestinal physiological functions and neural signaling, thereby maintaining the balance of the immune system and metabolism. However, disruptions to the gut microbiota can occur due to trauma, poor lifestyle habits, or antibiotic misuse, which can adversely affect the body’s homeostasis and contribute to the development of various diseases (77). In addition to their inherent neurogenic bowel dysfunction, patients with SCI often suffer direct damage to the autonomic neural circuits as a result of chronic psychological stress and abrupt lifestyle changes (78). This may lead to reduced gastrointestinal nervous system activity, prolonged emptying time, gastrointestinal motility disorders, fecal incontinence, constipation, and even gastrointestinal atrophy (79, 80). Therefore, analyzing changes in gut microbiota following SCI to develop appropriate intervention strategies holds significant clinical value for enhancing the quality of life of SCI patients.

Dietary interventions can directly influence the gut microbiota and metabolites in patients with SCI by affecting nutrition and energy balance (81). There exists a bidirectional relationship between SCI and constipation; while SCI can induce constipation, the latter may also contribute to gut flora dysbiosis, which impairs nutrient absorption and hinders neurological recovery. SCI induces alterations in the abundance of gut bacteria, including *Bifidobacterium*, *Bacteroides*, *Roseburia*, and *Lactobacillus*. Clinical studies have demonstrated that in constipated patients treated with *Lactobacillus plantarum P9*, beneficial bacteria such as *Lactobacillus plantarum* and *Ruminococcus gnavus* were significantly enriched. This intervention notably enhanced the metabolism of amino acids (such as L-asparagine and L-pipecolic acid) and short/medium-chain fatty acids (including valeric acid and caprylic acid), thereby alleviating symptoms of constipation (82). Long-term supplementation with *Bifidobacterium longum BB536* has shown similar effects (83). Consequently, improving constipation through probiotic supplementation represents a potential therapeutic target for SCI.

Patients with SCI often require frequent admissions to the Intensive Care Unit, prolonged mechanical ventilation, and high-dose antibiotic therapy. Additionally, they may experience neurogenic bowel dysfunction, placing them at an elevated risk for intestinal colonization by multidrug-resistant bacteria (MRB), which complicates treatment strategies (84). In a study conducted by Jiri Kriz et al. (85), FMT was utilized to infuse healthy donor feces through a nasoduodenal tube into SCI patients colonized with MRB, specifically carbapenemase-producing Enterobacteriaceae and vancomycin-resistant Enterococci. The findings indicated that FMT can restore gut microbiota diversity, thereby inhibiting MRB pathogen colonization and promoting overall recovery in patients. Importantly, the post-FMT administration of antibiotics may diminish the success rate of MRB decolonization; thus, it should be minimized in clinical practice.

SCI, particularly cervical and high thoracic injuries, impairs respiratory mechanics and leads to ventilator dependence, resulting in pulmonary infections, which are the primary cause of mortality in affected patients (86, 87). Yuanqing Ding et al. (72) observed that in patients with SCI, the abundance of intestinal *Bifidobacterium* was significantly reduced and exhibited a notable negative correlation with the enrichment of pulmonary *Enterobacteriaceae*. This suggests that *Bifidobacterium* deficiency is a core microbial characteristic of gut-lung axis dysregulation following SCI. Clinical association analyses indicate that *Bifidobacterium* can mitigate the incidence of pulmonary infection-related symptoms in SCI patients by maintaining intestinal microecological homeostasis, inhibiting the excessive proliferation of intestinal opportunistic pathogens, and reducing the translocation of gut-derived microbiota to the respiratory tract. This process reshapes the pulmonary microbiota structure and decreases the similarity between gut and lung microbiota. In a mouse model, oral administration of *Bifidobacterium longum BL300* significantly restored intestinal *Bifidobacterium* abundance and reshaped the intestinal microecological structure. It upregulated metabolic pathways related to short-chain fatty acid synthesis while downregulating pathogenic bacterial virulence and cell wall synthesis pathways. This intervention repaired the intestinal mucosal barrier, enhanced the expression of tight junction proteins ZO-1 and occludin, and protected the enteric nervous structure, thereby inhibiting the systemic translocation of bacteria and their products (LPS/LBP). *Bifidobacterium* significantly reduced the similarity between intestinal and pulmonary microbiota, as well as the colonization of gut-derived bacteria in the lungs of SCI mice. This led to a reduction in lung inflammatory infiltration, alveolar structural damage, and the abnormal activation of pro-inflammatory factors induced by Escherichia coli challenge. Ultimately, by stabilizing the crosstalk between the gut and lung axis, Bifidobacterium exerted a protective effect against pulmonary inflammation and injury following SCI. This finding provides a clinical basis for targeting the gut-lung axis to mitigate pulmonary complications after SCI.

A pilot crossover randomized controlled study involving 14 elite wheelchair athletes from Switzerland, with etiologies including spinal cord injury, spina bifida, and multiple sclerosis, employed a 12-week crossover design consisting of a 4-week multi-strain probiotic intervention, followed by a 4-week washout period, and concluding with a 4-week oat bran prebiotic intervention. The study found that the low inflammatory status of wheelchair athletes was significantly associated with higher alpha diversity in gut microbiota. The probiotic intervention was more effective in reducing levels of systemic inflammatory markers and significantly increased the species richness of gut microbiota, while also modulating its composition by enhancing the abundance of *Enterococcus* and reducing the abundance of pro-inflammatory bacteria. Furthermore, the probiotic intervention demonstrated a greater improvement in gut-related quality of life, with overall effects that surpassed those of the low-dose prebiotic intervention. These findings provide clinical evidence supporting the use of probiotic interventions to improve inflammatory status and gut microbiota balance in wheelchair athletes (88).

**Figure 1:**
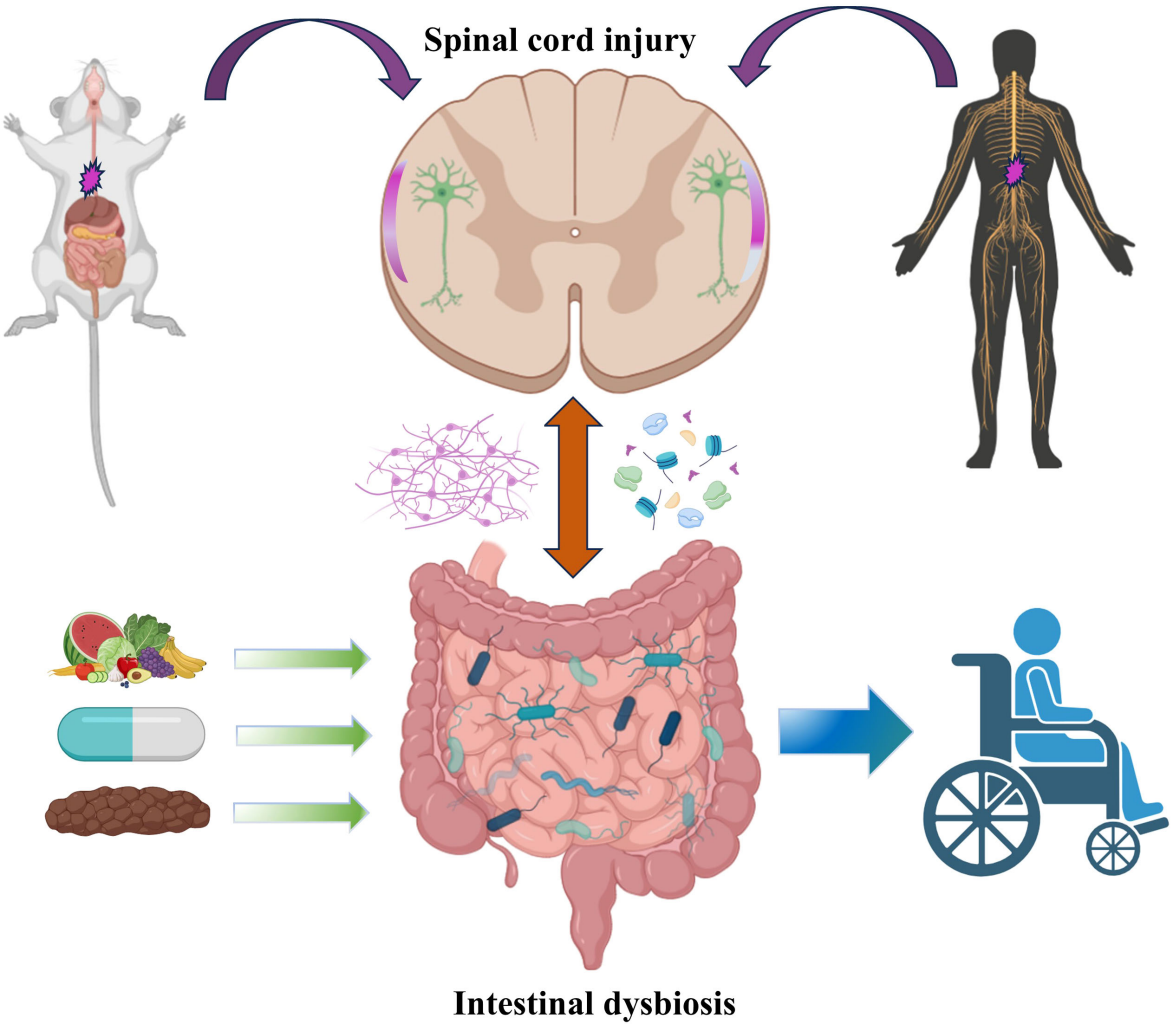
After spinal cord injury, there exists a mutual regulation among the spinal cord, microbiota, and gut axis. Dietary interventions, medications such as probiotics and fecal microbiota transplantation can all promote neural recovery by enhancing gut microbiota.

**Figure 2:**
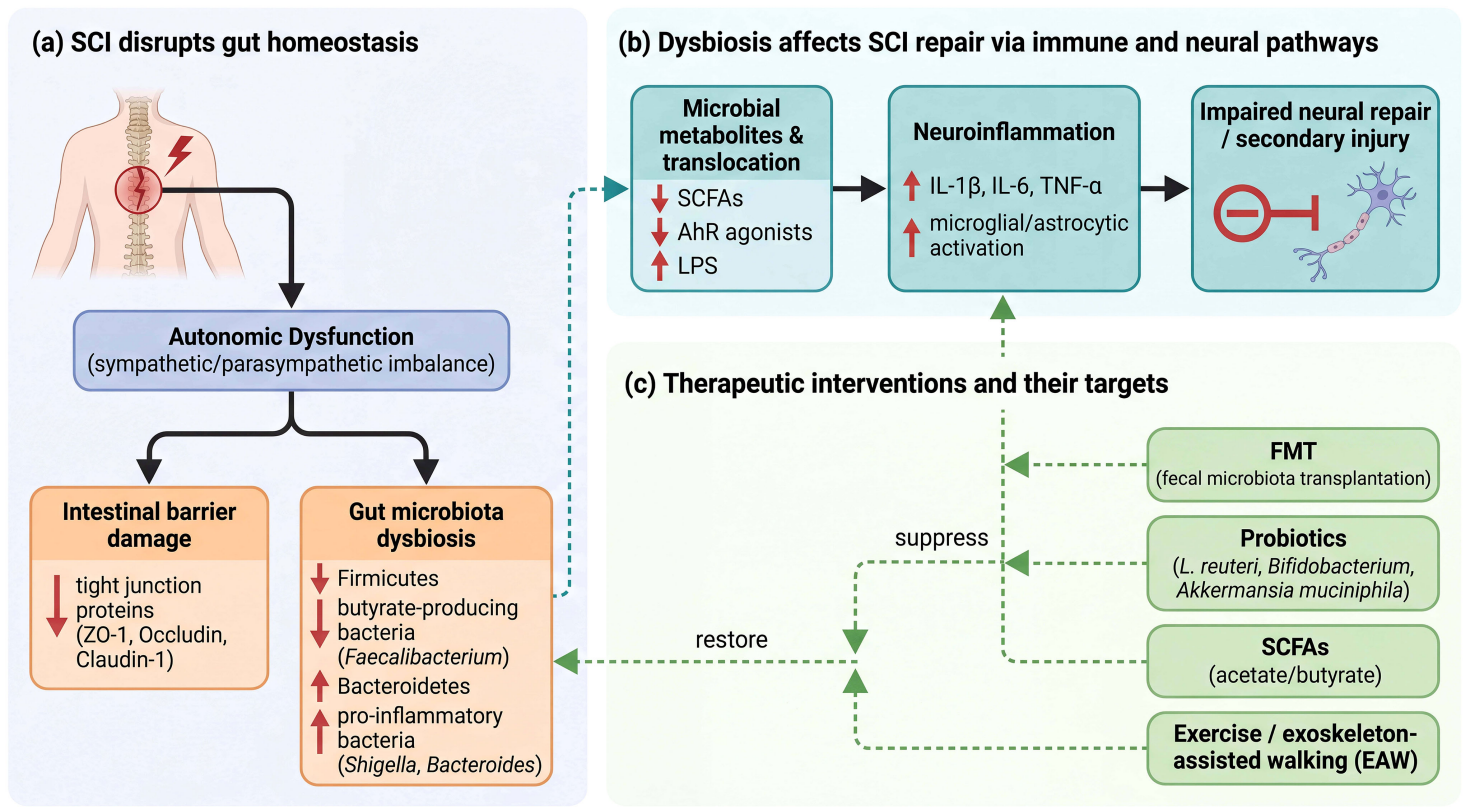
Bidirectional communication between spinal cord injury and gut microbiota and therapeutic interventions. **(a)** Spinal cord injury disrupts autonomic nervous system balance, leading to intestinal barrier damage and gut microbiota dysbiosis. **(b)** Gut dysbiosis promotes microbial metabolite imbalance, triggering neuroinflammation and impaired neural repair/secondary injury. **(c)** Therapeutic interventions including fecal microbiota transplantation, probiotics, SCFAs, and exercise/exoskeleton-assisted walking restore gut microbiota homeostasis and suppress neuroinflammation, potentially improving SCI recovery. AhR, aryl hydrocarbon receptor; EAW, exoskeleton-assisted walking; FMT, fecal microbiota transplantation; IL-1β, interleukin-1 beta; IL-6, interleukin-6; LPS, lipopolysaccharide; (L) reuteri, Lactobacillus reuteri; SCFAs, short-chain fatty acids; SCI, spinal cord injury; TNF-α, tumor necrosis factor alpha; ZO-1, zonula occludens-1.

Exercise is a significant intervention method that has been shown to enhance the abundance of beneficial bacterial genera, including *Blautia*, *Dialister*, and *Roseburia* (89), while also regulating intestinal motility, permeability, stool transit time, and consistency (90). Clinical trials have confirmed the beneficial effects of exercise on improving intestinal function and gut flora (91, 92). Furthermore, exoskeleton robots have become integrated into daily life and have been tested in clinical settings. The exercise training facilitated by exoskeleton systems has been utilized for the rehabilitation of paraplegic patients following SCI, demonstrating unique advantages in enhancing their intestinal function. Xiaomin Hu et al. (93) applied exoskeleton-assisted walking (EAW) to patients with complete motor paraplegia at the T2-L1 levels, revealing that EAW resulted in a decreased abundance of *Bacteroidetes* and *Verrucomicrobia*, while simultaneously upregulating the expression of *Firmicutes*, *Proteobacteria*, and *Actinobacteria* in SCI patients. Moreover, the abundances of *Bacteroides*, *Prevotella*, *Parabacteroides*, *Akkermansia*, *Blautia*, *Ruminococcus 2*, and *Megamonas* also exhibited a decline. Notably, the abundance of *Ralstonia* in the EAW group significantly increased. Additionally, EAW further alleviated symptoms of constipation and improved the quality of life for SCI patients. Currently, clinical studies focusing on the alleviation of neurological damage through interventions targeting the gut microbiota of patients are limited, indicating a need for further in-depth exploration A summary of the clinical investigations is provided in **Table 2**. The mutual regulatory mechanism of the gut-spinal cord axis is shown in **Figures 1**, **2**.

**Table 2:** Clinical studies.

Therapeutic methods	Types of spinal cord injuries	Number of cases	Changes in microbiota	Mechanism of action	Ref
FMT	Motor complete traumatic spinal cord injury with infection by MRB strain	7	Suppressed: Colonization of Klebsiella pneumoniae, Escherichia coli, vancomycin-resistant Enterococcus, or multidrug-resistant Acinetobacter baumannii	FMT can eliminate the colonization of MRB	(85) Jiri Kriz et al., 2024
EAW	Complete motor paraplegia at the T2-L1 levels	20	Bacteroidetes↓, Verrucomicrobia↓, Bacteroides↓, Prevotella↓, Parabacteroides↓, Akkermansia↓, Blautia↓, Ruminococcus 2↓, Megamonas↓ Firmicutes↑, Proteobacteria↑, Actinobacteria↑, Ruminococcus 1↑, Ruminococcaceae UCG002↑, Faecalibacterium↑, Dialister↑, Ralstonia↑, Escherichia-Shigella↑, Bifidobacterium↑	EAW may improve intestinal function and reduce injury in SCI patients to a limited extent by altering gut flora abundance	(93) Xiaomin Hu et al., 2024
Bifidobacterium	Iatrogenic spinal cord injury	14	Pulmonary (sputum): Streptococcus↑, Enterobacterales↑, Lactobacillales↑, Enterobacteriaceae↑, Bacilli↑, Prevotella↓, Prevotella↓ Intestinal: Bifidobacterium↓	Bifidobacterium maintains gut-lung axis homeostasis by inhibiting pathogenic Enterobacteriaceae overgrowth and reducing gut-derived bacterial translocation, thereby lowering pulmonary infection risk	(72) Yuanqing Ding et al., 2026

EAW, Exoskeleton-assisted walking; FMT, Fecal microbiota transplantation; MRB, Multidrug-resistant bacteria.

Despite encouraging preliminary clinical effects achieved by probiotic supplementation, FMT, dietary adjustment and rehabilitation exercise in regulating gut microbiota and alleviating SCI-related complications, multiple translational obstacles remain to be resolved. Uniform criteria for selecting optimal probiotic strains tailored to heterogeneous SCI patients are still lacking. As most SCI patients are immunocompromised, the safety risks and standardized management of FMT require further clarification, and concurrent antibiotic use also limits its clinical efficacy. Moreover, gut microbial metabolites have not been sufficiently validated as reliable clinical biomarkers for disease assessment and therapeutic monitoring. Additionally, most existing clinical investigations are small-sample pilot studies, and well-designed large-scale randomized controlled trials with unified intervention protocols and long-term outcome evaluation are urgently needed to accelerate the transformation of preclinical findings into standardized clinical strategies for SCI treatment.

## 6 Conclusions and future perspectives

Rehabilitation following spinal cord injury is a multifaceted process, wherein the ‘spinal cord-microbiota-gut’ axis may play a pivotal role in regulating neural repair post-injury. The sympathetic and parasympathetic nerves, which originate from the lateral horn of the spinal cord, innervate the gut, forming a relatively autonomous enteric nervous system that governs gastrointestinal function and sustains microbiota homeostasis (94, 95). Spinal cord injury disrupts intestinal motility and neural regulation, leading to dysbiosis of the gut microbiota and immune dysfunction. Factors such as motor impairment, anxiety, dietary and nutritional changes, chronic post-injury pain, and frequent antibiotic usage further exacerbate these effects (24, 34). The regulatory interaction between the spinal cord and the gut is bidirectional; autonomic dysfunction can precipitate dysbiosis of the gut microbiota following traumatic spinal cord injury, resulting in alterations in microbial diversity and abundance. This dysbiosis manifests at multiple levels, including bacterial phyla, families, and genera, and is correlated with inflammatory responses post-injury. Potential contributors to dysbiosis include neurogenic bowel dysfunction due to severe autonomic dysfunction following spinal cord injury (24), intestinal barrier impairment leading to gut microbiota translocation (11), and trauma-induced changes in nutrient intake and absorption (96).

The gut microbiota can influence the spinal cord nervous system through its metabolites and derivatives. Among these, AhR agonists produced by microbial metabolism (97), SCFAs (24), and microbiota-derived lipopolysaccharides play a crucial regulatory role in central nervous system inflammation. The exacerbation of secondary inflammatory responses following spinal cord injury may be linked to reduced levels of AhR agonists derived from microbial metabolism in spinal cord tissues (98). Moreover, SCFAs produced by gut microbiota metabolism can modify intestinal epithelial cell permeability and serve as significant energy substrates, improving electrolyte imbalances in the body and exerting substantial regulatory effects on central nervous system stability and inflammatory responses. Furthermore, in the central nervous system, SCFAs promote the conversion of Th17 cells into Treg cells, thereby suppressing inflammatory responses (99, 100). The gut microbiota has garnered considerable attention as a potential intervention strategy. Currently, probiotics and microbiota transplantation are employed in clinical treatments (101, 102). Conversely, the direct supplementation of microbial metabolites (such as AhR agonists and SCFAs) also represents a promising therapeutic approach for spinal cord injury. However, there are currently limited clinical studies on gut microbiota-based treatments for spinal cord injury. This remains a primary research focus for future work, aiming to provide more precise therapeutic targets for spinal cord injury treatment and complication prevention.

To advance this emerging field, future investigations should shift from descriptive correlation analyses to in-depth mechanistic and translational explorations. First, comprehensive multi-omics strategies, including metagenomics, metabolomics and transcriptomics, should be extensively utilized to dissect the causal linkages between gut microbial dysbiosis, host immune response and neurological dysfunction, thereby uncovering core regulatory networks. Second, rather than relying on poorly characterized probiotic interventions or empirical fecal microbiota transplantation, the rational development of well-defined synthetic microbial consortia and standardized postbiotic formulations is urgently needed to improve safety, stability and clinical accessibility. Third, high-quality clinical studies ought to systematically collect and integrate detailed patient metadata, such as age, comorbidities, medication history, dietary patterns and immune status, to conduct stratified subgroup analyses and reduce inter-individual heterogeneity. Fourth, current research mainly focuses on several classic microbial metabolites; hence, it is necessary to broaden the scope and explore the neuroprotective and immunomodulatory roles of underexamined microbial-derived metabolites. Collectively, these targeted strategies will facilitate the precise clinical translation of gut microbiota-based therapies and provide actionable directions for subsequent basic experiments and clinical trials.
